# Molecular epidemiology of novel swine origin influenza virus (S-OIV) from Gwalior, India, 2009

**DOI:** 10.1186/1743-422X-8-280

**Published:** 2011-06-07

**Authors:** Shashi Sharma, Manmohan Parida, Jyoti Shukla, PVL Rao

**Affiliations:** 1 Division of Virology, Defence Research & Development Establishment (DRDE), Gwalior- 474002, India

## Abstract

**Background:**

The H1N1pandemic virus is a newly emergent human influenza A virus that is closely related to a number of currently circulating pig viruses in the 'classic North American' and 'Eurasian' swine influenza virus lineages and thus referred as S-OIV. Since the first reports of the virus in humans in April 2009, H1N1 virus has spread to 168 countries and overseas territories. India also witnessed severe H1N1 pandemic virus epidemic with considerable morbidity and mortality in different parts starting from May 2009.

**Findings:**

The suspected swine flu outbreak from Gwalior India during October- December 2009 was confirmed through S-OIV HA gene specific RT-LAMP and real time RT-PCR. Positive samples through CDC real time and Lamp assay were further processed for isolation of the virus. Full HA gene sequencing of the H1N1 isolates of Gwalior, India revealed 99% homology with California and other circulating novel swine flu viruses. Three major changes were observed at nucleotide level, while two major amino acid shifts were observed at the position C9W and I30M corresponding to the ORF with prototype strain. The HA gene sequence phylogeny revealed the circulation of two genetically distinct lineages belonging to *Clade VII *and *Clade I *of S-OIV.

**Conclusions:**

Our findings also supported the earlier report about circulation of mixed genogroups of S-OIV in India. Therefore continuous monitoring of the genetic makeup of this newly emergent virus is essential to understand its evolution within the country.

## Findings

The Swine Flu is a respiratory viral disease which is usually found in pigs but can sometimes be transmitted to humans and cause epidemics or even pandemics (1). The viral strain involved is type A H1N1. The virus can be spread amongst humans from direct contact which can occur through coughing, sneezing or contamination of hands and surfaces. The severity of symptoms is highly variable, although with most people suffering only relatively mild symptoms (2). The pandemic that began in March 2009 was caused by H1N1 influenza A virus that had not been recognized previously in pigs or humans, although six of its eight gene segments were similar to ones previously detected in triple reassortant swine influenza viruses in pigs in North America (3). This strain represents a quadruple reassortment of two swine strains, one human strain, and one avian strain of influenza. The largest proportion of genes comes from swine influenza viruses (30.6 percent from North American swine influenza strains, 17.5 percent from Eurasian swine influenza strains), followed by North American avian influenza strains (34.4 percent) and human influenza strains (17.5 percent). Phylogenetic data even suggest that the reassortment of swine lineages may have occurred years before emergence in humans (4). Surprisingly however, there has been no evidence so far that pigs have played any role in the epidemiology or in the worldwide spread of the virus in human populations.

The pandemic H1N1 virus was first detected in India in May 2009 (5). Since then outbreaks have been reported from many parts of the country. As of December 6, 2009 the total number of confirmed cases in India was 19,632 with 621 deaths. Other reports comparing the HA gene sequence with those of the earlier influenza pandemics have shown that human-specific markers supporting efficient transmissibility of these viruses in human are present in the H1N1pdm virus (6). A recent study revealed that the early diversification of the H1N1pdm virus based on concatenated whole genomes resulted into seven lineages, Clade *I-VII*, that showed defined spatial patterns (7). A focal outbreak of swine flu was reported from Military Hospital Gwalior (Madhya Pradesh), India during October-December 2009. The detail molecular investigation of this Swine Flu outbreak including the genetic relatedness of this local isolates with regard to Indian and global isolates is reported.

## Methods

A total of 40 nasopharyngeal swab samples were collected with informed consents from suspected patients admitted to military hospital, Gwalior. The samples were collected with Hiviral™nylon flocked swab in Hiviral™ transport medium and were transported immediately to the laboratory. Initially RNA was extracted from 140 μl of sample using QIAmp viral RNA minikit (Qiagen, Germany) according to the manufacturer's specification.

All these samples were investigated by WHO approved CDC recommended real time RTPCR assay in 25 μl reaction scale using a panel of oligonucleotide primers and dual labeled hydrolysis probe employing ABI One-step RT-PCR kit (8). All the 40 samples were also simultaneously subjected to one step real-time reverse transcription loop mediated isothermal gene amplification (RTLAMP) assay for rapid and real-time detection of novel H1N1 Swine Flu virus RNA in clinical specimens by targeting the HA gene (9). The gene amplification in RTLAMP was accomplished by incubating the reaction mixture at 63°C for 60 min in routine laboratory water bath/dry heating bath. The real-time monitoring of RT-LAMP amplification was observed through spectrophotometric analysis by recording O.D. at 400 nm at every 6 second with the help of loop amp real-time turbidimeter (LA-200, Teramecs, Japan).

The Real time RT-PCR positive and RT-LAMP positive Swine flu clinical samples that were collected during recent 2009 outbreak from Gwalior were subjected to virus isolation. The nasopharyngeal swabs were filtered through 0.2 μm syringe filters and were passaged in Madin Darby Canine Kidney (MDCK) cells along with PBS as negative control (10). All the samples were processed under BSL-3+ laboratory. The sequencing of HA gene was performed with automated ABI Prism 3130 instrument (Applied Biosystems, Foster City CA, USA) by use of Big Dye3.1 cycle sequencing kits provided by the same manufacturer. Subsequently, any unincorporated labeled dNTP's were removed using Dye-X removal Centrisep column purification kit (Applied Biosystem). The nucleotide uences were edited and assembled using DNASTAR Lasergene 5 software package (Bioinformatics Pioneer DNASTAR, Inc., Wisconsin, USA), and the sequences are subjected to BLAST to find closest possible match from available sequences in GenBank at http://www.ncbi.nlm.nih.gov/BLAST. Multiple sequence alignments were carried out employing CLUSTALW version 1.83 (11). The phylogenetic analysis was done employing Neighbour-Joining (NJ) and Maximum-Parsimony (MP) methods using MEGA4 software (12). The tree was constructed using the Kimura's two-parameter distance model with 1000 bootstrap replicates. The percent nucleotide identity (PNI) and percent amino acid identity (PAI) values were calculated as pair wise p-distances.

## Results

The first isolate from India (A/India-Hyd/NIV51/2009) was from a traveler reaching Hyderabad on May 13, 2009 from the USA. Positive cases of H1N1pdm virus were thereafter detected from major cities (Pune, Delhi, Mumbai, Chennai and Bangalore) with maximum fatality reported from Pune and Bangalore (5).

All the clinical specimens having continuous fever, shivering, cough, with history of traveling history and closed contact with confirmed patients. All samples were processed for virus isolation.

The infected culture fluid (ICF) was collected after observation of virus specific cytopathic changes like clumping of cells, granulation, syncitia formation, finally detachment from the flask surface were observed. The presence of the virus in infected culture fluid was confirmed through ELISA using the HA antibody *(Sigenics, USA) *as well as by S-OIV HA gene specific RTLAMP and SYBR Green I real time RTPCR using H1N1 HA specific primers F3/B3 (Genomic Position 529-547/699-716).

The HA gene target was amplified by WHO recommended primers. All sequences have been submitted in Gen Bank with the following accession numbers (Accession No - GU 265731, GU 265730, GU265729, GU 201599, GU201598).

**Figure 1:**
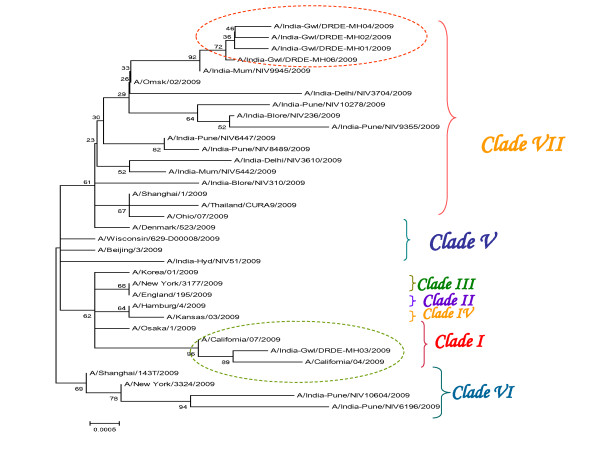
**HA Phylogeny of 5 Indian isolates with respect to circulating global isolates**. Scale bar indicates number of nucleotide substitutions per site. Each strain is abbreviated with country of origin and year of isolation.

The partial HA gene of sequences of 30 other representative pdmH1N1 virus of diverse geographical origins were retrieved from GenBank (Table-1). The phylogenetic analysis of the partial HA gene sequences revealed 99% homology at nucleotide level with novel swine flu virus. The BLAST analysis revealed 99% similarity with California 2009 prototype strain of novel swine flu virus. The sequence analysis of these Indian isolates revealed specific mutation at nucleotide level and amino acid changes respectively with respect to 22 global H1N1 pandemic isolates including few Indian isolates. Among the isolates, a sequence homology of 99.9% was observed. Three major changes were observed at nucleotide level, while two major amino acid shifts were observed at the position C9W and I30M corresponding to the ORF with prototype strain. Phylogenetic analysis based on partial HA gene classified the geographical diverse pdm H1N1 virus isolates into seven distinct clades (Figure 1).

## Discussion

Diversity of these Indian isolates based on partial HA gene phylogeny revealed the co-circulation of Clade *VII *and Clade *I *with predominance of Clade *VII *as already reported from Southern India. Multiple lineages of influenza A viruses were found to co-circulate during any single season and to undergo frequent reassortment. This, in turn, has had a major impact on antigenic evolution. Our study also further supported the fact that pandemic (H1N1) 2009 virus has evolved worldwide, shifting from an initial mixed clade pattern to the predominance of one Clade (Clade *VII*) during the course of the pandemic. The hypothesis that Clade *VII *virus enjoyed a marked advantage, in terms of transmissibility, over other early Clades is intriguing, but has yet to be demonstrated. The virus constituting this Clade was therefore responsible for most of the pandemic burden worldwide. In summary, this is the first report from central region of India (Madhya Pradesh) regarding the emergence, isolation and characterization of novel swine flu virus.

## Competing interests

The authors declare that they have no competing interests.

## Authors' contributions

All authors read and approved the final manuscript. MMP has designed the study plan and the experiments were executed by SS and JS. JS has carried out the sample analysis by RTLAMP while SS has performed the real-time RTPCR, RTPCR and sequence phylogeny. PVLR has supervised the study plan and gave critical suggestions for preparing the MS.

## Author's Information

Ms. Shashi Sharma has been working for last 3 years in the field of molecular epidemiology of circulating dengue virus genotypes.
